# Meniscal Regenerative Scaffolds Based on Biopolymers and Polymers: Recent Status and Applications

**DOI:** 10.3389/fcell.2021.661802

**Published:** 2021-07-13

**Authors:** Hao Li, Pinxue Li, Zhen Yang, Cangjian Gao, Liwei Fu, Zhiyao Liao, Tianyuan Zhao, Fuyang Cao, Wei Chen, Yu Peng, Zhiguo Yuan, Xiang Sui, Shuyun Liu, Quanyi Guo

**Affiliations:** ^1^The First Medical Center, Chinese PLA General Hospital, Institute of Orthopedics, Beijing, China; ^2^Beijing Key Lab of Regenerative Medicine in Orthopedics, Beijing, China; ^3^Key Laboratory of Musculoskeletal Trauma and War Injuries PLA, Beijing, China; ^4^School of Medicine, Nankai University, Tianjin, China; ^5^Department of Bone and Joint Surgery, Renji Hospital, School of Medicine, Shanghai Jiao Tong University, Shanghai, China

**Keywords:** polymeric scaffold, natural polymer, synthetic polymer, meniscal tissue engineering, meniscal regeneration

## Abstract

Knee menisci are structurally complex components that preserve appropriate biomechanics of the knee. Meniscal tissue is susceptible to injury and cannot heal spontaneously from most pathologies, especially considering the limited regenerative capacity of the inner avascular region. Conventional clinical treatments span from conservative therapy to meniscus implantation, all with limitations. There have been advances in meniscal tissue engineering and regenerative medicine in terms of potential combinations of polymeric biomaterials, endogenous cells and stimuli, resulting in innovative strategies. Recently, polymeric scaffolds have provided researchers with a powerful instrument to rationally support the requirements for meniscal tissue regeneration, ranging from an ideal architecture to biocompatibility and bioactivity. However, multiple challenges involving the anisotropic structure, sophisticated regenerative process, and challenging healing environment of the meniscus still create barriers to clinical application. Advances in scaffold manufacturing technology, temporal regulation of molecular signaling and investigation of host immunoresponses to scaffolds in tissue engineering provide alternative strategies, and studies have shed light on this field. Accordingly, this review aims to summarize the current polymers used to fabricate meniscal scaffolds and their applications *in vivo* and *in vitro* to evaluate their potential utility in meniscal tissue engineering. Recent progress on combinations of two or more types of polymers is described, with a focus on advanced strategies associated with technologies and immune compatibility and tunability. Finally, we discuss the current challenges and future prospects for regenerating injured meniscal tissues.

## Introduction

The importance of the meniscus in knee homeostasis has been widely acknowledged; unfortunately, meniscus-related injurie are quite common. According to the epidemiologic data reported by Logerstedt et al. (2010), the incidence rate of meniscus injury was 12–14%, and the prevalence was 61 cases per 100,000 persons. In the United States, injuries to the menisci are the most common injury to the knee, and 10–20% of all orthopedic surgeries involve surgical procedures to the meniscus (Montgomery et al., 2013). Nearly one million meniscal surgeries are conducted annually in the United States, most of which consist of partial or total meniscectomy, and the total cost for inpatient stays ranges from $500 million to $5 billion (Jones et al., 2012; Fillingham et al., 2017). Of the two genders, men were the most likely to experience meniscal tears, with reported ratios between 3:1 and 4:1 (Murphy et al., 2019).

Given the severe physical and psychological burden on individuals and the socioeconomic burden brought about by meniscal injuries, studies on the treatment and pathology of meniscal injuries are worthwhile endeavors. Meniscal tears, similar to many other musculoskeletal diseases, mainly occur in sports-related activities (32%) and non-sports-related activities (38%) and can arise from any specific event (28%) (Drosos and Pozo, 2004). As a result of the combination of axial impact forces and rotational forces between the femoral condyles and the tibial plateau, shear force may cause acute and degenerative tears of the meniscus, and these injuries are more likely to occur in the medial meniscus (Twomey-Kozak and Jayasuriya, 2020). Tears occurring in the inner avascular region of the meniscus are commonly complex and thorny and are often associated with a poor prognosis after surgical repair (Makris et al., 2011). In addition to the symptoms and motor dysfunctions, knee osteoarthritis (OA) is a common pathological response to meniscal injury (Englund et al., 2012). Briefly, meniscal degeneration or meniscectomy results in consistent articular cartilage overloading, leading to the development of OA (Katz et al., 2013). Therefore, preserving as much meniscal tissue as possible has become a widely prevailing trend (Crema et al., 2010).

Recently, considerable efforts have been put into meniscal regeneration rather than meniscal resection. The complex array of meniscal tissue structure and avascularity presents quite a thorny problem for clinicians; thus, tissue engineering aimed at tissue remodeling and functional restoration seems to be an alternative strategy (Twomey-Kozak and Jayasuriya, 2020). From traditional allograft menisci to currently used polymer materials, tissue-engineered meniscus scaffolds have been continuously progressing. First, the primary goal of meniscal tissue engineering is to develop a bioartificial substitute presenting the same level of components and architectures as native menisci. The design of structural-composition biomimetic scaffolds based on natural polymers, synthetic polymers, and a combination of multiple polymers has demonstrated fascinating meniscal regenerative capabilities. Furthermore, one major concern regarding successful meniscus regeneration was that how the polymeric scaffold reconstructs the zonal difference in the red and white zones of the meniscus. Generally, the tissue-engineered scaffold should mimic the zone-dependent arrangement of collagen fibers, ECM composition, and different bioactive inducers. In recent, intensive researches have been developed via advanced techniques and achieved promising repairing results. Therefore, we will subsequently focus on the recent development of various kinds of polymeric scaffolds in meniscal repair, with additional attention paid to reproduce zonal variations of meniscus.

Second, despite the importance of tissue-engineered scaffolds with characteristics that recapitulate the structure and composition of the meniscus, there are still many obstacles for the use of current materials to recreate a natural inducive microenvironment close to native meniscal tissue (Chen M. et al., 2019). The general design criteria of novel polymeric scaffolds are thus to recreate the main properties of the native microenvironment in terms of microarchitecture, components, and pro-regenerative features in order to stimulate cellular growth and maintain cell phenotype (da Silva Morais et al., 2020). Among several processing technologies, three-dimensional (3D) printing is one of the most appropriate for meniscal scaffold construction due to its highly accurate control of scaffold microstructures and compositions, making it possible to meet the primary requirements of meniscal tissue engineering. In addition, polymeric scaffolds not only act as temporary templates for neotissue formation and integration but also interact with cells and bioactive factors to orchestrate tissue remodeling (Zhang and King, 2020). 3D printing has also been applied in drug delivery (Qu et al., 2021); ideally, with personalized 3D architectures and programmed drug release profiles, these engineered meniscal scaffolds are very promising for enhancing meniscal regeneration. Despite the advances of numerous polymeric biomaterials, the immunocompatibility and immunomodulation of meniscal grafts have not been developed and require further exploration. Clearly, all these important polymers and some advances in meniscal tissue engineering thus need to be introduced, along with a richer knowledge base in this field, so that we can design a biomaterial-based meniscal scaffold that functionally recreates almost all of the aspects needed.

In this review, the relevant polymers involved in meniscal repair and regeneration are presented after a brief introduction to the anatomy, biochemical content, cells, and biomechanical properties of the meniscus, as well as a summary of conventional therapies. Then, we provide an overview of the different polymers and relevant scaffolds studied to date, with particular attention given to discussing the strategies reported recently on how to construct hybrid scaffolds to achieve versatile functions. Subsequently, we introduce additive manufacturing technologies used to promote the meniscus *in vitro* and *in vivo*, the recent advancement on zonal meniscal reconstruction and the effect of the applied biopolymers on the immune response and tissue regeneration were also discussed. Finally, we describe the main challenges and future development directions in advancing meniscal regeneration approaches.

## Meniscus Anatomy, Physiology, and Conventional Treatments

### Meniscus Anatomy and Cellular Components

**FIGURE 1:**
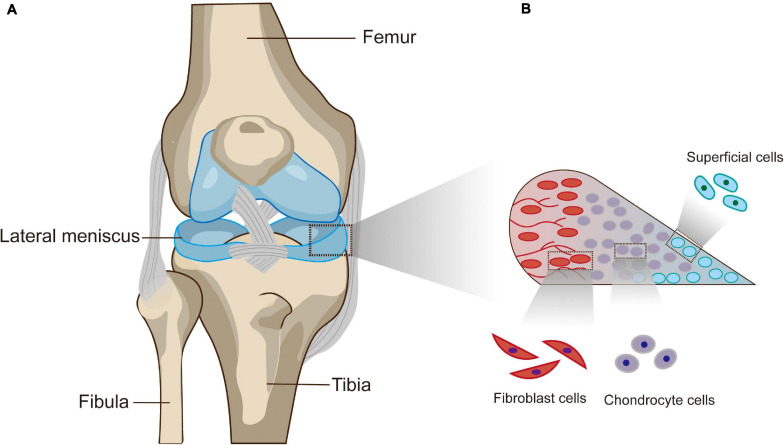
**(A)** Basic anatomy of the knee. **(B)** Cross-sectional diagram of the meniscus.

The meniscus is a pair of crescent, wedge-shaped fibrocartilaginous pads located between the femoral condyle and tibial plateau that serve a variety of functions, such as distributing loads, absorbing shock, maintaining stability, and contributing to cartilage lubrication and nutrition (Makris et al., 2011; Rongen et al., 2014). From a macroscopic point of view, the medial and lateral menisci possess their own anatomical variations, but the anterior horns of both are connected by the anterior intermeniscal ligament (Rongen et al., 2014; Figure 1A). Since vascularization of the meniscus decreases as the meniscus matures, the limited healing capacity of the inner zone of the meniscus is directly related to the poor blood supply, and nutrients can only be received from passive synovial fluid diffusion (Arnoczky and Warren, 1982; Petersen and Tillmann, 1995; Makris et al., 2011). Microscopically, it is reasonable to distinguish the peripheral red zone from the inner avascular white zone (Murphy et al., 2019). The inner zone is characterized by chondrocyte-like cells embedded in collagen type II and glycosaminoglycans (GAGs). In contrast to the inner zone, the peripheral zone presents abundant collagen type I deposition and many more elongated fibroblast-like cells (Makris et al., 2011; Jacob et al., 2020). In addition, the cells within the superficial zone are postulated to produce and secrete lubricant and anti-adhesive proteins or act as progenitor cells with regenerative potential (Lee et al., 2008; Jacob et al., 2020; Figure 1B).

### Meniscus Mechanical Properties and Functions

Generally, the meniscus plays a critical role in maintaining normal knee joint mechanics and functions. A number of studies have been performed to measure the mechanical strength of meniscal tissue in humans (Table 1). The specific mechanical properties of the meniscus are mainly determined by highly spatially oriented collagen fibers (Rongen et al., 2014). Indeed, the most important role of the collagen-proteoglycan meniscal matrix is its capacity to provide mechanical support, such as resistance to tension, compression and shear stress (Fithian et al., 1990). Specifically, the meniscus transfers 50–90% of the joint reaction forces under weight-bearing conditions (Walker and Erkman, 1975; Ahmed and Burke, 1983), with load transfer and absorption occurring via well-characterized mechanisms. In general, circumferential stresses within meniscal tissue are generated after joint surface contact, transferring compressive loads into horizontal tensile stress. Excessive energy being absorbed by collagen can also be released via the expulsion of synovial fluid (Storm et al., 2005; Rongen et al., 2014). Other important functions of the meniscus include providing lubrication, supplying nutrients to the cartilage and supporting proprioception (Jacob et al., 2020).

**TABLE 1:** Native meniscal tissue physical characteristics.

**Human meniscus**		Tensile properties (Tissakht and Ahmed, 1995)		Compressive properties (Sweigart et al., 2004)	
		Average radial tensile modulus (MPa)	Average circumferential tensile modulus (MPa)	Average aggregate modulus (MPa)	Average permeability [10^–15^ m4/(N⋅s)]
Medial	Anterior	6.01	91.23	0.16	1.78
	Central	10.47	76.82	0.11	1.54
	Posterior	12.73	81.14	0.10	2.03
Lateral	Anterior	9.03	108.27	–	
	Central	12.52	103.62		
	Posterior	13.36	123.09		

### Meniscus Pathologies and Conventional Therapies

In addition to trauma, other risk factors affect meniscal tissue, such as genetic susceptibility, obesity and knee malalignment (Englund et al., 2012). Twisting or shearing motions with a varus or valgus force account for the mechanism of most meniscal tears (Pihl et al., 2017). For younger patients, acute traumatic injury is a major cause of meniscal tears, and as in elderly patients, degenerative meniscal tears might act as key factors in the development of knee OA (Englund et al., 2007, 2012). The healing capacity of the meniscus after injury is basically dictated by the tear pattern and location. For instance, horizontal and radial tears involving the inner zone are thought to have the least healing potential owing to incursion into the avascular inner zone (Kwon et al., 2019). Unfortunately, meniscal injury is often followed by knee OA, which is known as the “meniscal pathway.” Briefly, loss of meniscal mechanical support leads to dramatically increased structural stress on articular cartilage, causing loss of cartilage, subchondral bone changes, and bone marrow lesions (Englund et al., 2012). In addition, the subsequent increased proinflammatory state within the knee joint after meniscal tears contributes to the progression of OA (Bigoni et al., 2017).

Conventional therapies for meniscal tears include both non-surgical and surgical approaches (Li et al., 2020a). Arthroscopic meniscectomy is the most commonly used surgical procedure for meniscal injuries (Katz and Martin, 2009; Kim et al., 2011b). However, it inevitably results in progressive cartilage degeneration and OA, and the curative effect on degenerative meniscal tears remains a matter of debate (Fairbank, 1948; Roos et al., 1998; Monk et al., 2017). Meniscal allograft transplantation (MAT) may further restore knee function, but this advantage is countered by the disadvantages of the insufficient number of donors and risk of disease transmission, immune rejection and non-matching (Noyes and Barber-Westin, 2010; Noyes and Barber-Westin, 2016; Parkinson et al., 2016). Chondroprotective evidence also needs to be validated (Vrancken et al., 2013). In search of a clinical solution for meniscal injury and joint homeostasis restoration, tissue engineering and scaffold-based regenerative medicine strategies have become some of the most promising approaches (Bandyopadhyay and Mandal, 2019). In this context, this review focuses on details of the polymeric aspects of meniscal therapy and advanced, novel polymeric scaffold-based strategies for meniscal repair and regeneration.

## Polymeric Scaffold-Based Strategies for Meniscal Regeneration

### Various Factors Involved in Polymeric Scaffold-Based Strategies

Tissue engineering techniques often involve the application of scaffolds, cells and biochemical and biomechanical stimuli to create engineered tissues (Kwon et al., 2019). These three main components collectively form many combinations and have obtained some promising advances. Therefore, to better understand the interaction among these three main components, cells and physical and biochemical signals all need to be introduced. Cells are important players in meniscal tissue engineering. Stem/progenitor/multipotent cell sources in meniscal tissue engineering can be obtained from various tissues, including bone marrow, synovium, and adipose tissue (Bilgen et al., 2018). Another large family of cells originates from mature connective tissue, such as the meniscus and cartilage (Peretti et al., 2004; Zellner et al., 2017). Biochemical stimuli also play an important role in engineering meniscal scaffolds. To increase extracellular matrix (ECM) production in engineered meniscal tissue, the administration of biochemical stimuli, such as growth factors, has long been used. A variety of growth factors, including platelet-derived growth factor (PDGF), bone morphogenetic protein-2 (BMP-2), transforming growth factor-β (TGF-β), insulin-like growth factor-1 (IGF-1), and fibroblast growth factor (FGF), have shown efficacy in improving meniscal regeneration (Bhargava et al., 1999; Pangborn and Athanasiou, 2005; Gunja and Athanasiou, 2010; Puetzer et al., 2013). Changes in oxygen tension have also yielded mixed effects on engineered meniscal tissue, which showed improved ACAN and COL2A1 expression under hypoxic conditions (Liang et al., 2017). On the other hand, the development of biomechanical stimuli for meniscal tissue engineering has focused on replicating heterogeneity and matrix-level arrangement of the tissue (Kwon et al., 2019). For example, compression and hydrostatic pressure have been used to improve the functional properties of meniscal neotissue (MacBarb et al., 2013; Zellner et al., 2015; Puetzer and Bonassar, 2016).

### Consideration of Polymeric Scaffold Design

Generally, scaffold design is of pivotal importance to accelerate meniscal tissue repair and regeneration. Polymer selection and biophysical and biochemical properties all need to be taken into consideration when designing an optimal tissue-engineered meniscal scaffold.

**FIGURE 2:**
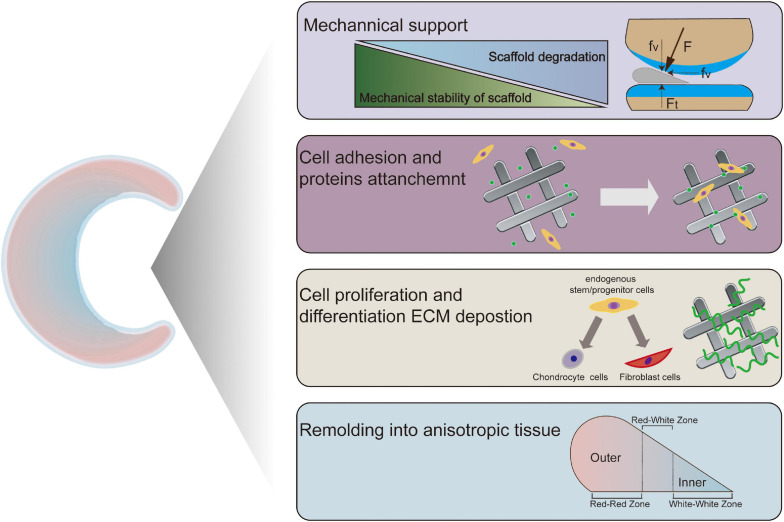
Key steps controlled/promoted by tissue-engineered meniscal scaffolds.

It is well known that biocompatibility and bioactivity are major considerations that may lead to scar tissue formation if not achieved (Murphy et al., 2018). In meniscal tissue engineering, a microenvironment conducive to cell adhesion, cell proliferation and matrix synthesis is necessary (Tan and Cooper-White, 2011). Natural ECM is a sophisticated 3D network that can support cells and control cellular responses, such as migration, proliferation and differentiation, via autocrine and paracrine mechanisms (Murphy et al., 2018; Subbiah and Guldberg, 2019). Therefore, compositionally, the scaffold needs to create an ECM-mimicking microenvironment with biocompatibility and minor immune rejection and degrade into harmless products along with meniscal tissue growth. The biophysical properties of natural ECM can also modulate cell behaviors (Elliott et al., 2019; Gaharwar et al., 2020). In regard to the architecture, the anisotropic orientation as well as suitable pore size and porosity are required to provide an optimal structure for cell ingrowth. In addition, scaffolds are also required to have appropriate mechanical properties, which enable the scaffold to preserve the normal contact biomechanics of the knee. In summary, biomaterial design and fabrication should mimic the biomechanics and components of natural ECM in meniscal regeneration (Ma, 2008; Zhou and Lee, 2011). Furthermore, the processing techniques should be convenient and versatile enough for clinically customized application. The specific design criteria of meniscal scaffolds are summarized in Table 2. To apply polymeric scaffold-based regenerative strategies in the context of the meniscus, clarification of the key role of scaffolds in meniscal tissue remodeling and maturation is needed. Collectively, a polymeric scaffold should not only provide a supportive microenvironment but also favor the migration, proliferation and differentiation of meniscogenic cells (Figure 2). In this review, we will summarize recent developments in polymeric scaffolds in terms of compositions, structures, processing technologies and bioactivities.

**TABLE 2:** Scaffold design consideration for effective meniscal tissue regeneration.

Requirement	Description
Biocompatibility	Low immunogenic response and toxicity of degradation products
Biodegradability	Coordinated degradation rate with host tissue regeneration
Biomechanical properties	Ability to withstand high cyclic loads, exceeding or matching the mechanical strength of natural meniscal tissue
Suitable porosity	Sufficient porous architecture and interlinked channels suitable for cell filling, metabolism, and efficient transfer of nutrients and wastes
Bioactivity	Capability of maintaining chemical stimuli to accelerate tissue ingrowth
Tunable properties and processibility	Ease of fabrication and clinical manipulation, resistance to long-term creep deformation

## Polymers for Meniscal Tissue Engineering Applications

Researchers consistently utilize natural and synthetic polymers or their combinations as engineered scaffolds and have demonstrated their promising properties for meniscal regeneration (Murphy et al., 2018). The advantages and limitations of commonly used polymers for meniscal tissue engineering are summarized in Table 3. Considering their excellent biocompatibility, processability and ECM-mimicking cues, natural polymers, such as collagen, silk fibroin (SF), and chitosan, present defined advantages for tissue engineering but are restricted by poor mechanical properties and non-tunable degradation (Prabhath et al., 2018; Tong et al., 2020). Therefore, synthetic polymers with favorable mechanical properties, simple fabrication methods and predictable degradation have been used as alternatives. However, synthetic polymers suffer from low cell affinity and require modification by biomolecules to improve their bioactivity (Makris et al., 2011; da Silva Morais et al., 2020). Moreover, the biodegradation of polymers is of the utmost importance since the degradation rate needs to be tuned in accordance with the required initial mechanical support, sustained drug delivery and space for neotissue formation (Engineer et al., 2011; Prabhath et al., 2018). In addition, numerous studies have been conducted to investigate the therapeutic effects of hybrid polymeric scaffolds, which combine the advantages of two or more natural and synthetic polymers, enabling the realization of comprehensive defined biophysical properties and bioactivity.

**TABLE 3:** Most relevant natural and synthetic polymer types used in meniscal tissue engineering.

Polymeric materials		Types	Advantages	Limitations	References
**Natural**	**Proteins**	Collagen	Cytocompatibility Capable of clinically use	Immunogenicity Weak mechanical strength	Patel et al., 2019
		Gelatin	Biocompatibility Biodegradability	Unfavorable mechanical properties	Echave et al., 2017; Zarif, 2018
		SF	Flexible processability Biocompatibility Capable of chemical modification Thermal stability Good mechanical strength	Immunogenicity Poor cell adhesion	Donnaloja et al., 2020; Li et al., 2020a
	**Polysaccharides**	Agarose	Controllable self-gelation properties Adjustable mechanical properties Non-immunogenic properties	Low cell adhesion	Zarrintaj et al., 2018; Salati et al., 2020b
		HA	Enzymatic biodegradability Viscoelasticity Capable of inducing chondrogenesis Chemically modifiable	Low mechanical properties Short degradation time	Kim et al., 2011a; Donnaloja et al., 2020; Silva et al., 2020
		Alginate	Biocompatibility Abundant source Easy gel formation	Poor cell attachment Difficult sterilization	Lee and Mooney, 2012; Ogay et al., 2020
		Chitosan	Biocompatibility Biodegradability Bioadhesion Easy physical and chemical functionalization	Long gelation time Short *in vivo* degradation time	Levengood and Zhang, 2014; Patel et al., 2019; Ogay et al., 2020
**Synthetic**	**Aliphatic polyesters**	PGA	Excellent mechanical properties Bioresorbability	Potential adverse tissue reaction for polymer fragments	Gui et al., 2011; Cojocaru et al., 2020
		PLA	High mechanical strength Thermal stability Tunable properties	Acidic products Autocatalytic degradation	Gregor et al., 2017; Patel et al., 2019
		PLGA	Tunable degradability Biocompatibility	Acidic byproducts	Lü et al., 2009; Zhou et al., 2012
		PCL	Biocompatibility Biodegradability	Hydrophobicity Limited cellular interaction	Abedalwafa et al., 2013; Mondal et al., 2016; Silva et al., 2020
	**Others**	PEG	Cytocompatibility Hydrophilicity Non-immunogenicity Biodegradability	No inherent functional groups	Patel et al., 2019; Ogay et al., 2020
		PU	Excellent mechanical properties and cytocompatibility Thermoplasticity	Long-term duration	Bharadwaz and Jayasuriya, 2020
		PCU	Flexibility Biocompatibility Biostability	Potential host tissue fusion failure in orthopedics	Abar et al., 2020
		PVA	Biocompatibility Bioadhesion Non-toxicity Non-carcinogenicity Good forming ability Easy manufacturing capability	Low protein adsorption	Baker et al., 2012; Williams et al., 2015
		PEO	Limited cytotoxicity	Fast degradation	Kim et al., 2014
**Decellularized materials**		DMS/DMECM	Rich cell adhesion and biochemical cues	Poor mechanical strength Potential immunogenicity	Li et al., 2020a; Wang et al., 2020

*HA, hyaluronic acid; SF, silk fibroin; PGA, poly(glycolic acid); PLA, poly(lactic acid); PLGA, poly(lactic-co-glycolic acid); PCL, poly(ε-caprolactone); PEG, polyethylene glycol; PU, polyurethane; PCU, polycarbonate urethane; PVA, polyvinyl alcohol; PEO, poly(ethylene oxide); DMECM, decellularized meniscal extracellular matrix; DMS, decellularized meniscal scaffold.*

### Natural Polymers

Natural polymers, such as polysaccharides and proteins, are considered to have great potential for meniscal tissue engineering due to their excellent biocompatibility, processability and bioactivity (Murphy et al., 2018). These polymers are also characterized by some drawbacks, including limited tunability, uncontrollable degradation, undesirable immunogenicity and poor mechanical properties, and are thus susceptible to failure in meniscal repair and regeneration (Chocholata et al., 2019; Li et al., 2020a; Table 3).

#### Proteins

Collagen is the most prevalent component of the meniscus ECM (Murphy et al., 2018). In general, it has a triple-helix structure, forms a highly organized 3D architecture, and plays a crucial role in maintaining the biological and structural integrity of the ECM (Cen et al., 2008; da Silva Morais et al., 2020). Since collagen is the main component of hard tissues and fibrous tissue and has excellent biocompatibility and degradability and low antigenicity, a large number of tissue engineering studies have utilized it in scaffolds for orthopedic applications, such as those in bone (Sarkar et al., 2006), cartilage (Zhang et al., 2013), tendons (Caliari et al., 2011), and intervertebral discs (Wilke et al., 2006). Collagen is widely used in the construction of meniscal cartilage tissue engineering scaffolds in various manufacturing methods. Recently, Filardo et al. (2019) used magnetic resonance imaging (MRI) and 3D bioprinting technology to design and create a cell-laden, collagen-rich and bioengineered medial meniscal tissue model, which could help optimize the custom design of damaged meniscus implants. The application of collagen in electrospun scaffolds has also been investigated for meniscal regeneration. Baek et al. (2018) produced a multilayer structure consisting of a collagen type I scaffold and tricomponent gel (collagen type II, chondroitin, hyaluronan) loaded with different types of cells. This electrospun collagen scaffold was reported to be able to promote cell adhesion and proliferation and meniscus-like extracellular matrix secretion. With regard to clinical use, Marcheggiani Muccioli et al. (2020) recently presented a 10-year follow-up study on soccer players who had received arthroscopically implanted lateral collagen meniscus implants (CMIs). The results showed promising recovery of knee joint function, and obvious cartilage thinning was not observed on imaging (Marcheggiani Muccioli et al., 2020).

However, collagen scaffolds displayed poor mechanical properties and much faster degradation rates than scaffolds consisting of polysaccharides and synthetic polymers; thus, the combination of collagen with other natural polymers and biomolecules was investigated (Subbiah and Guldberg, 2019; da Silva Morais et al., 2020). For example, a collagen/hyaluronan-infused, 3D-printed polymeric scaffold for partial meniscus replacement showed enhanced mechanical properties that could simultaneously satisfy the requirements for resistance to axial compression and circumferential tension (Ghodbane et al., 2019).

As a modified and degraded form of collagen, gelatin is a natural polymer derived from the hydrolysis of animal collagen (Santoro et al., 2014; Aoki and Saito, 2020). Since the digestive process enables gelatin to lose the triple-helix structure of collagen and confers low antigenicity, high biocompatibility and convenient fabrication, it is widely used in tissue engineering (Van Den Bulcke et al., 2000; Hoque et al., 2015). Narita et al. (2012) incorporated fibroblast growth factor 2 (FGF-2) into a gelatin hydrogel and observed an increased meniscal cell density after its application in horizontal meniscal tears in rabbits. In another study, rabbit platelet-rich plasma (PRP) was impregnated into a freeze-dried gelatin hydrogel to repair a circular meniscal defect in the anterior portion of the inner zone. The results showed that PRP strongly enhanced the healing process of the avascular meniscal zone (Ishida et al., 2007). However, the disadvantages of rapid degradation and dissatisfactory mechanical properties limit the application of gelatin alone in meniscal regeneration. Correspondingly, gelatin could be functionalized with methacrylamide (GelMA) groups to enable photocrosslinking by UV, potentially with the assistance of photoinitiators (Van Den Bulcke et al., 2000). Thus, gelatin modified with GelMA has also been studied as a polymeric meniscal scaffold. A study showed that GelMA in the construct significantly enhanced the adhesion of chondrocytes and the secretion of type II collagen (Bahcecioglu et al., 2019, 2019a).

Silk is a natural protein fiber produced by insect larvae for cocoons, and *Bombyx mori* silkworm cocoons are the most predominant source of silk (Murphy et al., 2018; Kashirina et al., 2019). The silkworm cocoon is mainly composed of silk sericin (SS) and SF, and the latter possesses impressive mechanical properties, elasticity, favorable biocompatibility, low immunogenicity and predictable biodegradability (Altman et al., 2003; Guziewicz et al., 2011; Kashirina et al., 2019). SF sponges have been found to enhance energy absorption and protect chondrocytes due to their favorable elasticity and low interfacial shear force (Li et al., 2020d). A silk-based platform has already been used as a substitute for meniscectomy. Yan and coworkers optimized the combination of a silk sponge and collagen coating in a rabbit meniscectomy model by applying coated collagen internally and externally to enhance the biocompatibility and initial frictional properties, respectively. Silk-collagen composites induced the formation of more meniscus-like tissue and reduced cartilage wear (Yan et al., 2019). More recently, a PCL/SF/Sr^2+^ scaffold for total meniscal repair, whereby the scaffold was manufactured by 3D wet electrospinning, showed enhanced meniscal regeneration. The structural components and mechanical properties of the neomeniscus almost rivaled those of the native meniscus 6 months after implantation (Li et al., 2020b). Concerning the necessity of stable scaffold fixation to the meniscus, a study conducted by Cengiz proposed a highly interconnected, suturable scaffold composed of SF and 3D-printed poly(ε-caprolactone) (PCL) mesh. This composite porous scaffold improved the suture retention strength by up to 4-fold and exhibited favorable tissue infiltration and blood vessel invasion after subcutaneous implantation *in vivo* (Cengiz et al., 2019).

#### Polysaccharides

Among natural polymers, agarose represents a natural and neutral, transparent polysaccharide that has excellent water solubility, biocompatibility, tunable mechanical properties and controllable self-gelation properties (Salati et al., 2020a) and plays an important role in the inner region in meniscal tissue repair (Bahcecioglu et al., 2019, 2019a,b). Experiments have shown attractive GAG expression in the agarose-impregnated interior meniscal region. Dynamic compression under 10% strain was also confirmed to increase GAG production in agarose (Bahcecioglu et al., 2019,b). A mixture of agarose and GelMA hydrogel was found to induce aggrecan expression and produce a high ratio of collagen type II/collagen type I in human fibrochondrocyte-hydrogel constructs. Moreover, the construct consisting of the blended hydrogel combined with the PCL scaffold perfectly mimicked the natural meniscal interior region (Bahcecioglu et al., 2019).

Hyaluronic acid (HA) is a natural hydrophilic GAG that is found in connective tissue, such as cartilage ECM, and is especially abundant in synovial fluid (Zamboni et al., 2018; Shah et al., 2019). HA is capable of water absorption and retention and lubrication and is an ideal molecule for promoting cartilage formation (Kim et al., 2011a; Zhang et al., 2014). In addition, functional groups can be introduced to the backbone of HA to mediate the formation of crosslinked hydrogels (Collins and Birkinshaw, 2013). For example, Song et al. (2019) successfully fabricated a crosslinked methacrylated hyaluronic acid (MeHA) fibrous scaffold by methacrylate modification, followed by an electrospinning process. The soft and stiff fibrous mesh network was sandwiched between meniscal tissue, and subcutaneous implantation in athymic rats showed that the stiffer MeHA fibrous network exhibited more obvious cellular invasion and enhanced collagen deposition (Song et al., 2019). In addition, Murakami et al. (2019) evaluated the effects of HA on human inner and outer meniscal cells and found that cell migration and proliferation were both accelerated by HA in a concentration-dependent manner. This finding suggested the possibility of meniscal regeneration without the need for growth factors, as HA alone could inhibit apoptosis and promote cell migration and proliferation.

Alginate, obtained from brown algae, is an anionic polysaccharide that exhibits remarkably good scaffold-forming properties and is biocompatible, inexpensive and abundant (Lee and Mooney, 2012). Furthermore, alginate hydrogels can be crosslinked by various materials (e.g., Ca^2+^) for bioactive agents and cell delivery (Lee and Mooney, 2012; Venkatesan et al., 2015; Kim et al., 2019). By combining collagen, alginate (A) and oxidized alginate (ADA), Gupta’s group designed self-healing interpenetrating network (IPN) hydrogel-loaded scaffolds with dual crosslinking [Ca^2+^-based ionic crosslinking and Schiff base reaction crosslinking (A-A, A-ADA)] capabilities, which revealed great potential for supporting fibrochondrocyte behavior and chondrogenesis *in vitro* (Gupta et al., 2020a). Alginate has also been used in minimally invasive meniscal tissue engineering applications. For example, Kim et al. (2019) fabricated an ultrapurified alginate (UPAL) gel that was dicationically crosslinked by CaCl_2_ and injected into rabbit meniscal defects. The reparative tissues in the UPAL gel group had a mean stiffness of 27.8 ± 6.2 N/mm, which was significantly greater than that in the control group at 12 weeks (Kim et al., 2019). Other researchers have often used alginate in combination with other polysaccharides. Recently, Resmi et al. (2020) synthesized an injectable, self-crosslinking hydrogel from alginate dialdehyde and gelatin, and *ex vivo* application of this hydrogel in pig meniscal tears showed good integration with host meniscal tissue.

In contrast to alginate, chitosan is a linear, positively charged copolymer derived from deacetylated chitin, which can be found in the exoskeleton of fungal cell walls (Shah et al., 2019; Donnaloja et al., 2020). Chitosan has been extensively used in skin (Sandri et al., 2019), bone (Cui et al., 2019), cartilage (Izzo et al., 2019), and tendon (Depres-Tremblay et al., 2019) regeneration, as it demonstrates excellent biodegradability, and it is worth noting that the degradation rate can be regulated by the molecular mass and deacetylation degree (Vårum et al., 1997; Mi et al., 2002). A decellularized meniscal extracellular matrix (DMECM) and gelatin/chitosan (G/C) composite scaffold with a high elastic modulus and low cytotoxicity was reported by Yu et al. (2019). Chitosan has also been freeze dried in the fabrication of porous scaffolds for cell transplantation and tissue regeneration (Suh and Matthew, 2000). To further investigate the impact of the molar content of chitosan on the chondrogenic potential of mesenchymal stem cells (MSCs), a comparative study was performed. Chitosan (Ch) at different molar ratios was crosslinked with polyvinyl alcohol (PVA) using urethane prepolymer (PPU) chains, and articular chondrocytes (ACs) and adipose tissue-derived mesenchymal stem cells (ASCs) were then isolated and cultured. The authors confirmed that the AC-seeded PVA/Ch/PPU (1:4:1) scaffold showed higher expression levels of ECM components, superior meniscus regeneration and lower levels of cartilage degeneration than the comparator scaffolds (Moradi et al., 2017b).

### Synthetic Polymers

Owing to their poor mechanical strength, unstable degradation and limited sources, natural polymers are still insufficient for meniscal repair and regeneration. Therefore, synthetic polymers with favorable mechanical properties, reproducibility and controllable degradation have been widely used to produce meniscal scaffolds (Makris et al., 2011; da Silva Morais et al., 2020; Table 3). However, one of the limitations of synthetic scaffolds is their paucity of bioactive cues, which could be overcome by adding biological coatings (Silva et al., 2020).

As a biodegradable polymer with excellent biocompatibility and mechanical strength, poly(glycolic acid) (PGA) has been widely used in the biomechanical and medical fields since the 1970s and can serve as a scaffolding material to repair articular cartilage in clinical practice (Siclari et al., 2018; Otsuki et al., 2019; Cojocaru et al., 2020). In meniscal tissue engineering, a 3D, meniscal-like, PGA-hyaluronan implant with high porosity was developed, and this scaffold showed high biocompatibility and improved the expression of chondrogenic genes during coculture with human meniscal cells *in vitro*. In addition, in a sheep model, the scaffold showed greater proteoglycan and collagen type I production than the control group (Cojocaru et al., 2020). For *in vivo* evaluation, a meniscus-shaped scaffold made of PGA covered with a polylactic acid/caprolactone [P(LA/CL)] sponge was implanted into the right knee in a medial meniscus resection minipig model. The results showed that the scaffold provided appropriate initial strength and could prevent cartilage degeneration with relatively low inflammation. However, the compressive stress and elastic modulus of the scaffold were significantly inferior to those of the native meniscus (Otsuki et al., 2019).

Poly(lactic acid) (PLA) is a thermoplastic aliphatic polyester obtained from the polymerization of lactic acid and/or the ring-opening polymerization of lactide, which has suitable biocompatibility and biodegradability but also some drawbacks, such as a high cost, long degradation time, low utility and limited molecular weight (Castro-Aguirre et al., 2016; Murariu and Dubois, 2016). In an interesting study, core-shell coaxial nanofibrous scaffolds were prepared by electrospinning. The PLA core provided mechanical strength, while the collagen shell facilitated cellular adhesion and matrix synthesis. *In vivo* experiments showed excellent integration between the scaffold and native tissue (Baek et al., 2019). The filamentous PLA structure was printed by 3D printing and modified by surface modification with active functional groups. Then, IPN hydrogels populated with hMSCs were applied to the surface-modified PLA structure for *in vitro* and *in vivo* research. At 28 days after implantation in a rat model, the structure of PLA remained relatively intact, which was consistent with the degradation curve *in vitro*. The integrity of the PLA scaffold ensured minimal mechanical stress on the hMSCs, allowing optimal function to be achieved (Gupta et al., 2020a).

**FIGURE 3:**
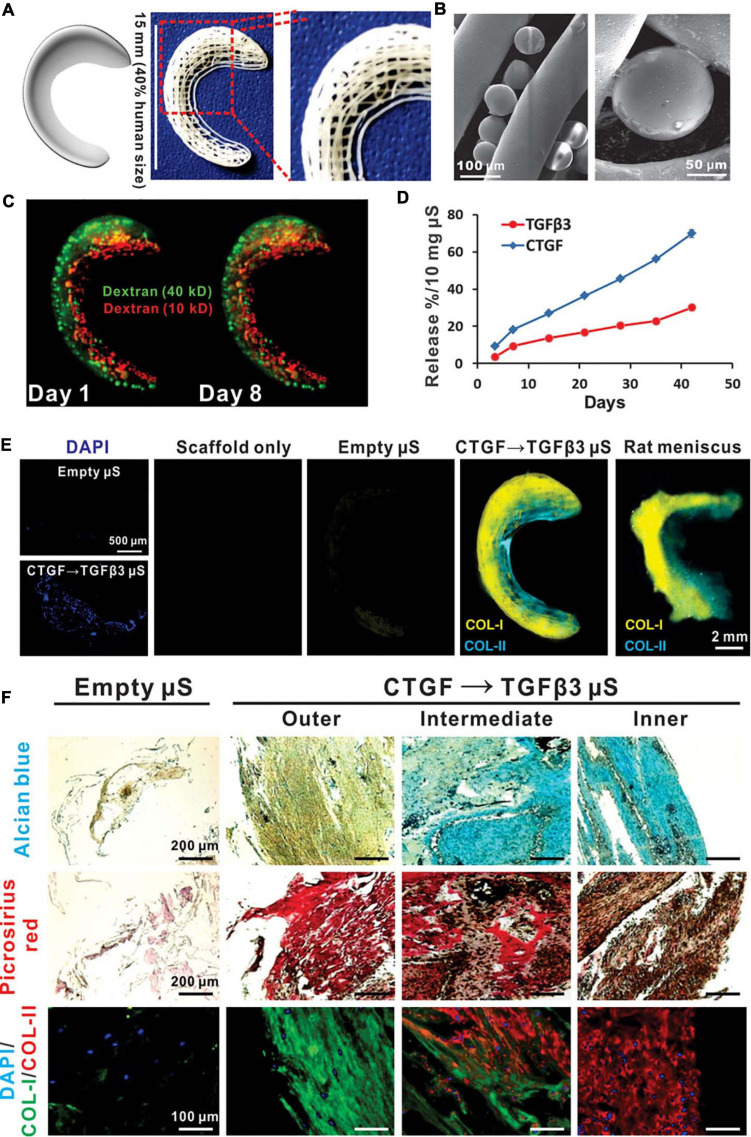
Spatiotemporally released rhCTGF and rhTGF-β3 induced fibrocartilage-like matrix formation in 3D-printed, porous scaffolds. **(A)** Anatomical reconstruction of human meniscus. Human meniscal scaffolds were 3D printed with the layer-by-layer deposition of PCL fibers (100 mm in diameter), forming 100- to 200-mm channels. **(B)** PLGA microspheres (μS) encapsulating rhCTGF and rhTGF-β3 were in physical contact with PCL microfibers. **(C)** Fluorescent dextran simulating CTGF (green, 40 kD) and TGF-β3 (red, 10 kD) was delivered into the outer and inner zones, respectively, of human meniscal scaffolds to show scaffold loading. The distribution of dextran was maintained from day 1 to day 8. **(D)** rhCTGF and rhTGF-β3 release from the PCL scaffolds over time *in vitro*. **(E)** When the scaffolds were incubated atop human synovial MSC monolayers for 6 weeks, spatiotemporally delivered rhCTGF- and rhTGF-β3 induced cells to form zone-specific collagen type I and II matrices, similar to those in the native rat meniscus. **(F)** Scaffolds with empty mS showed little matrix formation after 6 weeks of coculture with a 1:1 mixture of fibrogenic and chondrogenic supplements (no growth factors in medium). Spatiotemporal delivery of rhCTGF and rhTGF-β3 induced fibrocartilaginous matrix formation, consisting of alcian blue-positive, collagen II-rich cartilaginous matrix in the inner zone and picrosirius red-positive, collagen I-rich fibrous matrix in the outer zone. A total of five replicates were tested, with representative images selected from the same scaffold (reprinted from Lee et al., 2014 with permission from AAAS).

Compared with that of PLA, the degradation time of poly(lactic-co-glycolic acid) (PLGA) can be controlled according to the glycolic acid content, while the higher the ethyl ester ratio is, the easier the polymer is to degrade (Zhou et al., 2012). PLGA is a linear copolymer composed of different proportions of glycolic acid and lactic acid monomers (Gentile et al., 2014). PLGA is an attractive polymer used in drug delivery and tissue engineering due to its favorable biodegradability, flexible processability, tunable degradation, surface functionalization and targeted drug delivery (Danhier et al., 2012; Mir et al., 2017; Zhao et al., 2021). PLGA is biodegradable because its ester linkages can be hydrolyzed in aqueous solution, and as byproducts, glycolic acid and lactic acid can be cleared from the body via normal metabolic pathways (Lü et al., 2009). Gu et al. (2012) used cartilage-derived morphogenetic protein-2 (CDMP-2) and TGF-β1 to preculture autologous myoblasts and then construct myoblast cell-seeded PLGA scaffolds, which presented accelerated healing after meniscal defect implantation. Regarding functionalized PLGA scaffolds, Kwak et al. (2017) reported a PRP-pretreated PLGA mesh scaffold seeded with human ACs to regenerate meniscal tissue. At 6 weeks after subcutaneous implantation, increased cell attachment and cartilaginous tissue formation were observed in the cell-seeded scaffold between native devitalized meniscal discs (Kwak et al., 2017). Other researchers have developed several PLGA-based approaches as drug delivery platforms. For instance, TGF-β3 and connective tissue growth factor (CTGF) loaded in PLGA microparticles were incorporated into 3D-printed PCL anatomical meniscal scaffolds and demonstrated promising reparative results (Lee et al., 2014; Figure 3).

PCL is a hydrophobic polyester with a low melting point (56–61°C), slow degradation, favorable compatibility and satisfactory mechanical strength (Abedalwafa et al., 2013; Teng et al., 2014). However, its hydrophobicity and inadequate wettability may lead to poor cell attachment and proliferation (Mondal et al., 2016; Silva et al., 2020). For that reason, the surface modification of PCL is crucial for its biological application (Mondal et al., 2016). In one study, galactose was incorporated into electrospun PCL nanofibrous scaffolds for meniscal cell culture, whereas the composite scaffold resulted in increased cell attachment and proliferation (Gopinathan et al., 2015). In addition, PCL has been used to prepare nanofiber scaffolds with a high effective surface area-to-volume ratio to facilitate the release of biomolecules and ensure greater interaction between seeded cells and biomolecules (Qu et al., 2019). For example, Qu et al. (2019) developed an aligned protein-containing scaffold based on electrospun PCL-PLGA fibers using bovine serum albumin (BSA) to stabilize the loaded TGF-β3. Compared with the high-dose TGF-β3-loaded scaffold, the low-dose TGF-β3-loaded nanofiber scaffold effectively activated the fibrochondrogenic differentiation of synovium-derived stem cells (SDSCs). The results indicated that fibrochondrogenesis and chondrogenesis differed by growth factor concentration, with the former requiring a lower dose (Qu et al., 2019).

Polyurethane (PU) possesses elasticity, thermoplasticity and excellent biocompatibility and has already been applied in meniscal tissue engineering (Venkatesan and Kim, 2014; Koch et al., 2018; Vedicherla et al., 2018; Bharadwaz and Jayasuriya, 2020). For example, Actifit^®^ implants have been studied as a cellular component delivery vehicle. Vedicherla et al. (2018) developed a cell-seeded PU scaffold. Fresh chondrocytes (FCs) and minced cartilage (MC) were cultured on the scaffold, and the tissue integration effect was evaluated in a caprine meniscal explant model. The results exhibited better matrix deposition and tissue integration in both the FC and MC groups than in the acellular scaffold group (Vedicherla et al., 2018). MSCs are also promising for meniscal repair due to their potential for fibrochondrogenesis and their ability to secrete reparative growth factors (Koch et al., 2018). An MSC-loaded PU scaffold was produced as a replacement for large, full-thickness meniscal defects. At 12 weeks after surgery, the vessel density in the scaffold group was superior to that in the cell-free groups. Additionally, significantly greater proteoglycan deposition and integration with the surrounding meniscal tissue were observed in the MSC-loaded group than in the acellular group. However, this advantage of MSC loading disappeared after 12 weeks (Koch et al., 2018). It has also been reported that MSC-seeded PU scaffolds exhibit little additional clinical benefit in the protection of articular cartilage (Olivos-Meza et al., 2019).

Another synthetic polymer that should be addressed is polycarbonate urethane (PCU). PCU is a flexible, biocompatible, biostable and wear-resistant material that can be incorporated in 3D-printed, porous structural scaffolds (Williams et al., 2015; Abar et al., 2020). In addition, as a hydrophilic material, PCU can mimic the lubrication mechanism in native synovial joints (Wan et al., 2020). A medial meniscus PCU prosthesis, called NUsurface^®^ (Active Implants Corp., Memphis, TN, United States), has been undergoing clinical trials and has become available on the market (Drobnič et al., 2019). Another novel meniscus-shaped, wear-resistant full implant made of PCU was also developed. The study showed that the posterior horn of the implant was under maximum pressure at 3 months, and the deformation at 12 months after implantation was acceptable. However, one implant failed due to a complete tear during posterior angular extension. Therefore, it is essential to strengthen the posterior horn of the implant to prevent fixation failure of one horn under extension. The damage progression in the implant group was similar to that in the allograft group but significantly worse than that in the non-operated group (Vrancken et al., 2017).

PVA is a polymer synthesized from partially or completely hydroxylated polyvinyl acetate (Baker et al., 2012). As a bioinert, non-carcinogenic, moist, biocompatible composite material (Hayes and Kennedy, 2016; Marrella et al., 2018), PVA possesses good formability, mechanical properties and manufacturability (Kobayashi et al., 2005; Moradi et al., 2017a; Marrella et al., 2018) and has been widely used in the field of regenerative tissue engineering. Polyvinyl alcohol hydrogel (PVA-H) has viscoelastic properties similar to those of cartilage and meniscal cartilage and does not wear out even after millions of compression cycles (Moradi et al., 2017a). As early as 2005, Kobayashi et al. (2005) developed an artificial meniscus with high-water-content (90%) PVA-H and conducted a preliminary study in a rabbit model. This study showed that the articular cartilage of the knee was still in good condition 2 years after the operation (Kobayashi et al., 2005). As a physically crosslinked gel, PVA-H does not contain toxic monomers that may be present after chemical crosslinking. However, its poor tensile properties limit its practical use, especially in strong fibrous tissues, such as the meniscus, tendons and ligaments (Holloway et al., 2010, 2014). Therefore, it is particularly important to modify PVA-H with new materials. A 3D biomimetic meniscal scaffold was designed using 3:1 SF/PVA. Autoclaved eggshell membrane (AESM) powder (1–3% w/v) was used as a biomechanical enhancer, and the composite scaffold presented with good load-bearing performance and improved meniscal tissue regeneration (Pillai et al., 2018).

In addition, various copolymers based on poly(ethylene oxide) (PEO) have been developed and applied in the field of drug delivery due to their good biocompatibility and fast, non-toxic degradation (Kim et al., 2014). PEO can be used as a sacrificial, water-soluble scaffold material to increase porosity and promote cell infiltration (Baker et al., 2008). As Qu’s group reported, collagenase-loaded PEO electrospun fibers may trigger matrix degradation. When applied in meniscal tears *in vitro*, PEO degradation was accompanied by the release of enzymes in the local wound edge and successfully increased both tissue porosity and cell migration (Qu et al., 2013).

### Decellularized Materials

The decellularization method has historically been used to isolate the ECM via cell removal and is widely applied in tissue regeneration (Gilbert et al., 2006). Due to their meniscus-specific chemical composition and architecture, implants derived from decellularized materials have been used as scaffolds in meniscal tissue engineering (Murphy et al., 2018; Ruprecht et al., 2019). Recently, decellularized materials have been explored for use in decellularized meniscal scaffolds (DMSs) to support the regeneration of meniscal tissue. For instance, Stabile et al. (2010) developed fresh-frozen meniscus allografts with increased porosity and promising mechanical integrity that presented potential for clinical application. DMECM presents components similar to those of the native meniscus and can be formed into various structures, thereby promoting cell infiltration and remodeling (Chen M. et al., 2019; Ruprecht et al., 2019). Researchers have demonstrated that meniscus-derived matrix scaffolds are capable of promoting the infiltration of endogenous meniscal cells and MSCs (Ruprecht et al., 2019). Although numerous studies have proven that decellularized matrix (DCM) may be able to regulate stem cell differentiation, Liang et al. (2018) demonstrated that synovial fluid-derived mesenchymal stem cell (SF-MSC)-loaded meniscus-derived DCM was incapable of inducing the differentiation of SF-MSCs into MFCs without TGF-β3 and IGF-1 supplementation. However, DCM materials suffer from poor performance in load-bearing applications. To tackle this problem, our team previously developed a hybrid scaffold for regenerating the meniscus in a rabbit model, combining acellular meniscus extracellular matrix (AMECM) and demineralized cancellous bone (DCB). The AMECM/DCB constructs demonstrated favorable mechanical properties and a promising capacity to promote fibrochondrocyte proliferation and GAG secretion (Yuan et al., 2016). Another common solution to improve the stiffness of implants is to hybridize them with other synthetic polymers. For example, Guo and coworkers used DMECM and a 3D-printed PCL scaffold to create a hybrid construct for rabbit meniscal regeneration. The hybrid scaffold displayed biomechanical strength similar to that of the native meniscus and facilitated whole meniscal regeneration in both rabbit and sheep meniscus repair models (Guo et al., 2021). Additionally, as another tissue engineering approach, DMECM-based injectable hydrogels have been developed. One early work by Yuan et al. (2017) developed hMSC-loaded DMECM hydrogels and found that the cell-laden DMECM hydrogel successfully retained the viability of hMSCs in nude rat meniscal injury for 8 weeks, resulting in neomeniscal tissue formation and preventing joint space narrowing, pathological mineralization and OA development.

### Hybrid Polymeric Scaffold for Meniscal Tissue Engineering

**FIGURE 4:**
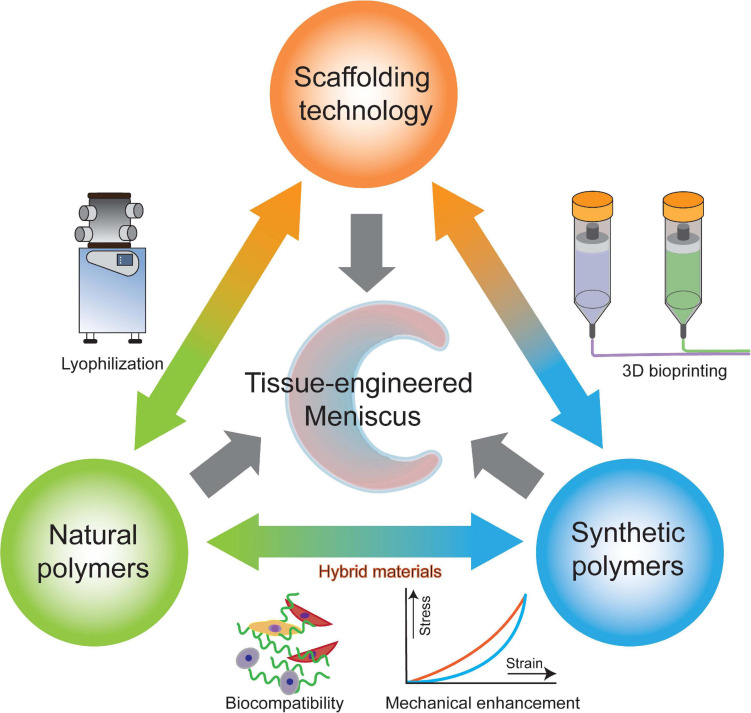
Key factors of polymeric scaffolds and their relationships.

Recently, researchers have fabricated numerous hybrid scaffolds made from two or more types of polymeric materials. While a single natural or synthetic polymer can only provide limited advantages for tissue-engineered meniscal scaffolds, the final product produced by a mixture of natural and synthetic polymers tends to possess comprehensive advantages that no single polymer can provide (Bakhshandeh et al., 2017). For example, natural polymers, such as chitosan, collagen and gelatin, usually contain biological molecules that can interact with cells, providing superior biological performance for hybrid polymeric scaffolds (Donnaloja et al., 2020), while synthetic polymers provide tunable physical properties such as mechanical support and a controllable degradation rate (Zhou et al., 2012; Figure 4).

Loading hybrid scaffolds with tissue-derived cells has the advantage of the encapuslated cells replenishing ECM loss and filling in defects as scaffold degradation occurs over time (Bilgen et al., 2018). For example, Chen M. et al. (2019) constructed a wedge-like, 3D-printed, MFC-loaded hybrid scaffold with a PCL scaffold as a backbone and then injected an optimized MECM-based Ca-alginate hydrogel (2%). *In vivo* experiments confirmed that the PCL-hydrogel-MFC group was similar to the sham group in terms of biochemical content, histological structures and biomechanical properties, which demonstrated an ideal capability for regeneration of the whole meniscus (Chen M. et al., 2019). In another study, by Cengiz et al. (2020), to meet the requirements of biomimetic meniscal scaffolds and cell coculture, PCL was blended with SF and entrapped in a 3D-printed cage (EiC) scaffold. Human stem cells or meniscocytes were cultured on the EiC scaffold and implanted subcutaneously in nude mice. The SF-based EiC scaffold showed better cell infiltration as well as a milder inflammatory response (Cengiz et al., 2020).

On the other hand, hybrid scaffolds combining polymers and biofactors can locally deliver signal molecules to create a favorable microenvironment. For instance, a PCL/SF hybrid scaffold based on 3D printing was developed by Li et al. (2020d) and exhibited a balance between the mechanical properties and degradation rate. The SF sponge provided cartilage protection due to its high elasticity and low interfacial shear force, the PCL provided excellent mechanical support, and the conjugation of a peptide with SMSC-specific affinity (LTHPRWP; L7) increased cellular recruitment and retention. *In vivo* experiments showed that this meticulously tailored scaffold greatly enhanced meniscal regeneration while protecting cartilage (Li et al., 2020d). In summary, hybrid scaffolds have been used for meniscal tissue engineering. The general design strategy consists of utilizing synthetic polymers as a supporting framework, with natural polymers more likely serving as an additive microenvironment to mimic extracellular microenvironments, while cells and bioactive factors may further assist in improving cell recruitment, proliferation, and differentiation and ultimately improve regeneration.

## Recent Advancements

### 3D Printing

Promising advances have been made in technology for the fabrication of meniscal scaffolds in terms of particulate leaching, freeze drying, solvent casting, electrospinning, and 3D printing (Esposito et al., 2013; Sun et al., 2017; Li et al., 2020e). Table 4 is a summary of the most relevant examples of meniscal scaffold fabrication techniques. Currently, in the fabrication of sponge scaffolds using physical and/or chemical treatments, the porous scaffold can provide a desirable microenvironment for the cells culturing, however, the pore size, porosity and interconnectivity cannot be controlled, and these scaffolds are often hindered by pore blockage effects *in vivo* (Sun et al., 2017). Another alternative manufacturing technology, electrospinning technology can be used to produce collagen-mimicking nanoscale fibers but is also limited in terms of controlling the structure and porosity of the construct (Megelski et al., 2002; Sun et al., 2017).

**TABLE 4:** Summary of the most relevant examples of meniscal scaffold fabrication techniques.

Scaffold fabrication methods	Polymeric materials	Biophysical properties [porosity (%), pore size (μm), mechanical properties)]	Cell types/growth factors	Animal models	Results	References
Lyophilization	PGA/HA	90% in the outer, 50% in the center 48.2 μm	Human meniscal cells	Partial meniscectomy sheep model	Favorable biocompatibility and increased expression of meniscus-related genes *in vitro* Repaired tissue rich in proteoglycans and type I collagen *in vivo*	Cojocaru et al., 2020
Lyophilization	DMECM/G/Ch	Elastic modulus (1% DMECM): 8.44 ± 0.11 MPa	Rat BM-MSCs	-	Improved cell proliferation, elastic modulus and cell viability *in vitro*, with no cytotoxicity	Yu et al., 2019
Lyophilization	PGA/P(LA/CL) sponge	Compressive modulus (50% strain): ∼5 MPa Tensile elastic modulus: ∼20 MPa	-	Partial medial meniscectomy mini pig model	Stimulated intrinsic and extrinsic regeneration and meniscal-like tissue formation	Otsuki et al., 2019
Blending Lyophilization	PVA/SF (reinforced and functionalized by AESM and UCM)	87.5% Mostly > 25 μm Compressive modulus: 26.7 ± 0.13 MPa Storage modulus: 14.8 MPa at 10 Hz Viscous modulus: 3.8 MPa	Human meniscal cells	Biotoxicity rabbit model	Supported cell proliferation and secretion of ECM components *in vitro* Angiogenesis, biocompatibility, and minimal inflammatory response *in vivo*	Pillai et al., 2018
Blending Crosslinking	PVA/Ch	120–160 μm Tensile modulus: 66.86 MPa (PVA) 110 MPa (PVA/Ch8)	Rabbit AD-MSCs and ACs	Total meniscectomy rabbit model	Ch4 scaffold seeded with ACs alone could repair meniscus *in vivo*, while scaffold seeded with AD-MSCs could not	Moradi et al., 2017a
Injection molding	PCU	Compressive modulus: 19.62 MPa	–	Total medial meniscectomy goat model	Maintained surface and geometrical integrity of the implant, with cartilage damage similar to that in the allograft group	Vrancken et al., 2017
3D wet-electrospinning	PCL/SF	Pore size: 100–200 μm Compressive modulus: 0.08 ± 0.02 MPa Tensile modulus: 12.6 ± 2.2 MPa	Rabbit AD-MSCs	Total medial meniscectomy rabbit model	Promoted cell migration and proliferation, increased secretion of ECM components *in vitro* Substantial formation of new meniscus-like tissue and protection of cartilage	Li et al., 2020b
Coaxial electrospinning	PLA/collagen	Tensile modulus: 376 ± 47 MPa	Human meniscal avascular cells	Young bovine meniscus explant model	Excellent mechanical strength Induced meniscogenic gene expression and generated meniscal tissue *ex vivo*	Baek et al., 2019
Coaxial electrospinning	PLA/PEG	Tensile modulus (10 mg/ml PEG): ∼35 MPa	Human meniscal and synovial cells	–	Addition of PDGF-BB enhanced meniscogenic gene expression *in vitro*	Baek et al., 2020
3D printing Crosslinking Lyophilization	PCL/SF	Compressive modulus: 6.582 ± 0.645 MPa Tensile modulus: 13.402 ± 3.119 MPa	Rat SM-MSCs	Total medial meniscectomy rabbit model	The scaffold provided a favorable microenvironment for SM-MSC migration, proliferation, differentiation, retention, and ECM production *in vitro* Enhanced the energy absorption ability of the meniscus and protected chondrocytes *in vivo*	Li et al., 2020e
3D printing Crosslinking	PLA/collagen-A-ADA	400 μm Young’s modulus: 130 MPa	Human UC-MSCs	*In vivo* biocompatibility rat model	Scaffold promoted proliferation, activity and differentiation of chondrocytes *in vitro* and showed promising biocompatibility *in vivo* after implantation	Gupta et al., 2020a
3D printing Blending	PCL/GelMA (outer region)/GelMA-Ag (outer region)	Pore size: 810 ± 40 μm Compressive modulus: 150–400 kPa Tensile modulus: 13–18 MPa	Human fibrochondrocytes	–	Cell-hydrogel constructs induced aggrecan expression and produced a high ratio of COL II/COL I *in vitro*	Bahcecioglu et al., 2019
3D printing Crosslinking	PCL/DMECM-A	> 80% >150 μm Compressive modulus: 6.54 ± 0.88 MPa Tensile modulus: 34.64 ± 2.34 MPa	Rabbit MFCs	Total medial meniscectomy rabbit model	Hybrid scaffold improved MFC proliferation and chondrogenic mRNA expression *in vitro* and showed regeneration of superior meniscus and articular cartilage protection *in vivo*	Chen M. et al., 2019
3D bioprinting	Collagen		Human BM-MSCs	–	Provided an anatomically shaped, patient-specific construct with good cellular biocompatibility	Filardo et al., 2019
3D bioprinting	PU/PCL/me-dECM	Compressive modulus: 3.49 ± 1.28 MPa	Human BM-MSCs	Total medial meniscectomy rabbit model	Scaffold exhibited favorable biocompatibility and excellent mechanical properties and promoted the formation of neofibrocartilage	Chae et al., 2021
3D bioprinting	PCL/gelatin/fibri nogen/HA/glycerol	–	Goat BM-MSCs	Total medial meniscectomy goat model	Improved mobility with minor articular cartilage degradation; the regenerated meniscus exhibited region-specific matrix components and cell phenotypes	Sun et al., 2020

*3D, three-dimensional; PGA, poly(glycolic acid); HA, hyaluronic acid; DMECM, decellularized meniscal extracellular matrix; G, gelatin; Ch, chitosan; PLA, polylactic acid; P(LA/CL), polylactic acid/caprolactone; PVA, polyvinyl alcohol; SF, silk fibroin; AESM, autoclaved eggshell membrane; UCM, unique combination of biomolecules; PCU, polycarbonate urethane; PCL, poly(ε-caprolactone); PEG, polyethylene glycol; A, alginate; ADA, oxidized alginate; GelMA, gelatin methacrylate; Ag, agarose; PU, polyurethane; me-dECM, decellularized meniscal extracellular matrix. BM-MSCs, bone marrow-derived mesenchymal stem cells; AD-MSCs, adipose-derived mesenchymal stem cells; ACs, articular chondrocytes; PDGF-BB, platelet-derived growth factor-BB; SM-MSCs, synovium-derived mesenchymal stem cells; UC-MSCs, umbilical cord-derived mesenchymal stem cells; MFCs, meniscal fibrochondrocytes.*

As an emerging additive manufacturing technology, 3D printing is able to produce components with ideal shapes and structures, allowing the accurate construction of specified 3D hierarchical structures (Bahcecioglu et al., 2019; Fu et al., 2020). Hence, among all of these scaffolding technologies, 3D printing is more promising in meniscal tissue engineering. Indeed, 3D bioprinting is a branch of the development of 3D printing technology and represents the technology’s entry into the field of tissue engineering and regenerative medicine (Matai et al., 2020). 3D bioprinting is an automated, tissue-friendly manufacturing method that very accurately simulates the actual arrangement of the cells and ECM of the targeted tissue, with the ability to construct the 3D tissue equivalent of the desired shape and structure, reproducing the complexity of human tissue (Murphy et al., 2018; Chae et al., 2021). Generally, bioprinting technologies mainly applied in meniscus/cartilage tissue engineering can be classified as follows: 3D plotting/direct ink writing, stereolithography (SLA), selective laser sintering (SLS), fused deposition modeling (FDM), and extrusion-based bioprinting (Moroni et al., 2018). Table 5 presents several commonly used bioprinting techniques and provides some comparative information. At present, there are many studies using FDM to produce a PCL framework and then combining hydrogels to fabricate composite meniscal scaffolds. However, FDM techniques suffer from poor surface quality and difficulties in combining biopolymers owing to their high extrusion temperatures (Agarwal et al., 2021). The SLA technique is characterized by high resolution; however, a limited selection of photopolymers restricts its application in tissue engineering, and it has not yet been applied in meniscal tissue engineering (Qu et al., 2021). Tissue-engineered meniscal scaffolds produced by extrusion bioprinting techniques have high yields and excellent structural integrity, and this technique is the most common 3D bioprinting technology in the field of meniscal regeneration.

**TABLE 5:** Traditional 3D printing techniques for meniscal tissue engineering.

	Process	Materials	Prons	Cons	References
Fused deposition modeling (FDM)	Heated polymer was extruded and hardened after printing to form solid construct	PCL (Chen M. et al., 2019) PLA (Gupta et al., 2020b)	Fast Convenient Repeatability Needs no support structure	High temperature Poor surface properties	Moroni et al., 2018; Agarwal et al., 2021
Extrusion-based Bioprinting (EBB)	Bioinks containing cells and biofactors were extruded as programmed and were crosslinked to form designed structures	PLA-PCL (Li et al., 2020e) SF/gelatin (Bandyopadhyay and Mandal, 2019) Collagen (Filardo et al., 2019) PU/PCL/me-dECM (Chae et al., 2021) PCL/gelatin/fibrinogen/HA/glycerol (Sun et al., 2020)	Direct deposition of cells and biomolecules	Low resolution Low mechanical strength Narrow material selection	Qu et al., 2021
Stereolithography (SLA)	Polymer solidified at focal points while exposed to focused light	GelMA, PEGDA, RNTK (Zhou et al., 2020) GelMA/methacrylated HA (Lam et al., 2019) GelMA/cECM (Chen P. et al., 2019)	High fabrication accuracy possibility to fabricate complex internal structures	Limited material selection	Corbel et al., 2011; Agarwal et al., 2021

*PEGDA, poly(ethylene glycol) diacrylate; RNTK, lysine-functionalized rosette nanotubes.*

From a polymeric materials science point of view, numerous polymers have been extensively investigated to serve as bioinks in 3D printing for tissue engineering. Bioinks, mainly hydrogels that contain cells and various biological components, play an important role in creating a compatible microenvironment for cellular activity (Roseti et al., 2018; Samadian et al., 2020). Some single-component polymer bioinks, such as SF (Bandyopadhyay and Mandal, 2019), alginate (Narayanan et al., 2018), and GelMA (Grogan et al., 2013) bioinks, have been used for 3D meniscal bioprinting. However, a single polymer cannot reproduce the complexity of ECM in natural tissue and thereby provide a good microenvironment for cells *in vivo*. Researchers have made immense efforts to develop an optimal bioink that simultaneously meets the requirements of biocompatibility and printability. Recently, tissue-specific meniscal dECM (me-dECM) bioinks based on 3D cell printing were designed by Chae et al. (2021) and helped preserve the complexity of the natural ECM, thus demonstrating the potential for tissue regeneration and special biological functions. This printable bioink supported the proliferation and differentiation of encapsulated stem cells, and induced fibrochondrocytes were also observed *in vitro* (Chae et al., 2021). In addition to combining hydrogels and solid polymers to mimic the biphasic structure of the meniscus and produce an optimal cellular microenvironment, 3D bioprinting has also been used to incorporate growth factors and cells in hydrogel-based scaffolds (Caterson et al., 2001; Sun et al., 2017). In a pilot *in vivo* study performed by Sun et al. (2020), a ready-to-implant anisotropic meniscal scaffold fabricated by 3D bioprinting was implanted in a goat meniscus transplantation model. This 3D-bioprinted meniscus substitute contained microspheres encapsulating CTGF and TGF-β3 and was mixed with BMSCs. The *in vitro* and *in vivo* results demonstrated cell phenotypes and matrix formation resembling those of the native meniscus, as well as chondroprotection of the goat articular cartilage (Sun et al., 2020).

In addition to the implications of the aforementioned traditional bioprinting techniques for the engineering of meniscus substitutes, some advanced techniques have also generated enthusiasm about their potential applications in meniscal tissue engineering. For example, the inkjet technique, which is suitable for printing cellularized scaffolds, can be used to print customized cellularized menisci. The process of inkjet printing usually consists of two parts: droplet formation on the target substrate and droplet interaction with related materials (Li et al., 2020c). Recently, in a proof-of-concept study, a cellularized human meniscus was produced by a microvalve-based inkjet technique. Primary human bone marrow mesenchymal stem cells were isolated, embedded with collagen bioink and customized inkjet printing, resulting in a patient-specific meniscal scaffold with superior anatomical structure and favorable cell compatibility (Filardo et al., 2019). In addition, some new bioprinting technologies based on non-contact (acoustoelectric, optical and magnetic) guidance of cell chemotaxis are gradually coming into play. Traditional scaffold technology guides the migration, adhesion and orientation of cells through the conformation of its own scaffold fibers and controls the expression of extracellular matrix according to the orientation of the fibers. A new acoustic electrophoretic three-dimensional (3D) biomanufacturing method, which uses radiation forces generated by superimposing ultrasonic bulk acoustic waves (BAWs) to preferentially organize arrays of cells in monolayer and multilayer hydrogel structures, was developed. The researchers investigated the effects of the parameters on the cell array configuration by controlling the frequency and amplitude of the ultrasonic wave, the signal voltage, the viscosity of the bioink, and the time of action. Finally, a physiologically related 3D construction of the meniscus was presented (Chansoria et al., 2019).

In conclusion, the excellent ability of 3D bioprinting technology to precisely control the fiber diameter, orientation, and microarchitecture endows the resulting constructs with promising mechanical properties and favorable biological functions. Therefore, it is one of the most attractive and promising technologies for tissue engineering applications, particularly meniscal regeneration.

### Meniscal Zonal Reconstruction Studies

Restoration the heterogeneities in anatomical, structural, mechanical and biochemical characteristics of meniscus is still a challenging goal albeit significant advances achieved in biomaterials science. Meniscal regeneration involves the two distinct regions that with different composition, structures and function. Hence, it requires an in-depth understanding of the zonal difference within meniscus and thus design such an optimal scaffold possessed with the anisotropic variation of (i) biomaterial composition, (ii) microarchitectures, and (iii) regional-specific pro-regenerative effects.

In this context, recent 3D fabrication techniques which can construct zone-dependent features, combined with polymeric biomaterials, have shed light on this topic. Firstly, biomimicry of meniscal spatial variation can be achieved by varying scaffolding materials. For example, in a study by Bahcecioglu et al. (2019), anatomically shaped PCL meniscal scaffolds were impregnated with cell-laden GelMA-agarose in the inner region and GelMA in the outer region. The *in vitro* results showed that inner GelMA-agarose hydrogel enhance chondrogenic differentiation, while the increasing COL I and decreasing COL II expression in the outer region was observed (Bahcecioglu et al., 2019). This structurally and biochemically correct scaffold presented with an advantageous solution on meniscus zonal reconstruction. Anatomical selected meniscus ECM were also exhibiting potential to develop anisotropic scaffolds able to induce the zonal fibrocartilage differentiation (Rothrauff et al., 2017). In addition to the biopolymers selections, the cell-loaded meniscal constructs in bioreactors is another approach to fulfill the zonal construction requirements. The application of bioreactors is commonly referred to applying controlled biophysical stimulus, especially valuable for hard tissue regeneration (Aguilar et al., 2019). Ideally, a zone-dependent mechanical stimulation in terms of tensile stimulus dominating on outer zone and compressive stimulus dominating on inner region can be a promising strategy for zonal differentiation (Puetzer and Bonassar, 2016). As an example, in Bahcecioglu et al. (2019a), fibrochondrocytes loaded in PCL scaffold were arranged to form scaffolds. The authors tested the effect of different cell-laden PCL/hydrogel using a custom-made bioreactor and compared the outcomes against static methods. The scaffolds treated with the dynamic stimulation resulted in increased ratio of COL II expression in the inner region (agarose impregnated along with compressive method), and enhanced ratio of COL I expression in the outer region (GelMA impregnated along with tensile method). From this instance, it is evident that bioreactor combined with spatial biopolymer incorporation enhance the formation of zone-specific tissues compared to the static seeding.

**FIGURE 5:**
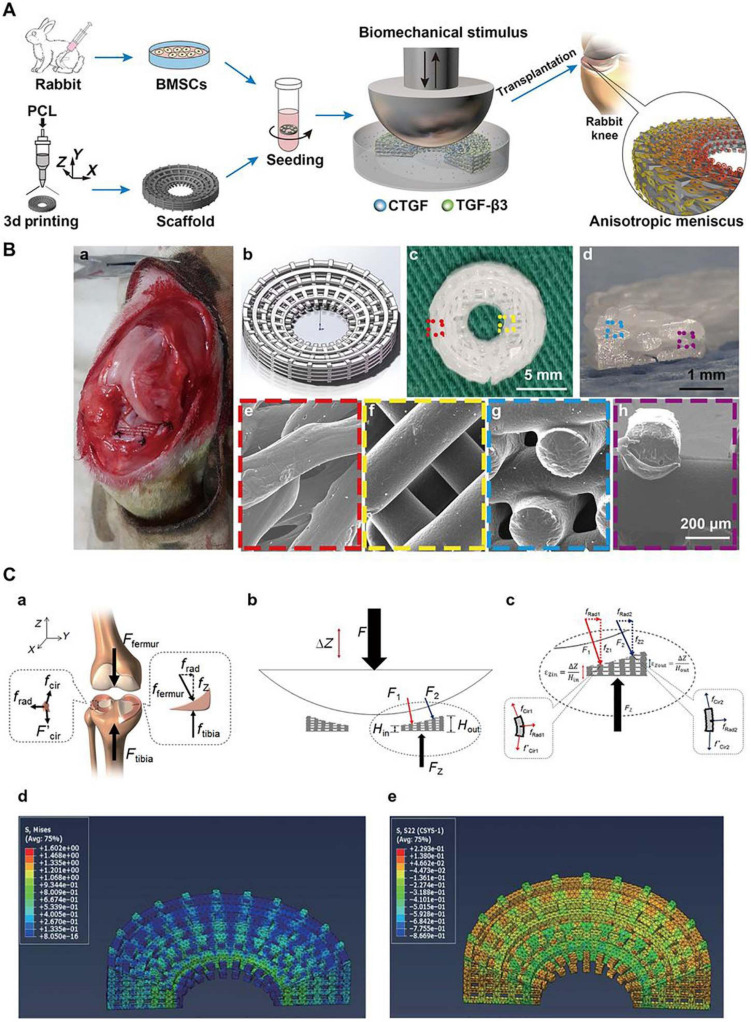
Schematic diagrams for the reconstruction of a functional anisotropic meniscus. **(A)** Flowchart of stem cell-based strategies for the construction of a tissue-engineered meniscus with an anisotropic structure. BMSCs, bone marrow-derived stem cells. **(B)** 3D-printed PCL scaffold for implantation. **(a)** Photograph of rabbit knee during implantation. **(b)** 3D scaffold design model. **(c,d)** Top and cross-sectional views of the wedge-shaped circular PCL scaffold. **(e,f)** Top views of the outer and inner regions of the PCL scaffold obtained by SEM. **(g,h)** Cross-sectional views of the outer and inner regions of the PCL scaffold obtained by SEM. **(e–h)** Higher magnification images of regions outlined in c and d. **(C)** Biomechanical simulation. **(a)** Forces typically transduced by the knee meniscus in the human body. **(b,c)** Applied loading forces across the meniscal construct. **(d,e)** Calculated stress fields across the meniscal construct at 10% displacement of the loading plate. **(d)** von Mises stress distribution with a gradual decrease in stress from the internal to external rings. **(e)** Compressive circumferential stress in the internal rings and tensile circumferential stress in the external rings (reprinted from Zhang et al., 2019 with permission from AAAS).

Furthermore, the region-specific infusion of various grow factors has also been explored for meniscal zonal reconstruction. For example, Lee et al. (2014) designed a 3D printed anatomic-mimicking PCL scaffold infused with different growth factors (Figure 3). The spatial-temporal releasing growth factors allowed dual induction of chondrogenic and fibrochondrogenic differentiation in the same construct (Lee et al., 2014). Another example is shown in Figure 5 where a dual growth factors functionalized scaffold was placed within a perfusion bioreactor (Zhang et al., 2019). The bioreactor was used to provide a consistent dynamic biomechanical stimulus to accelerate the spatial regulation of fibrochondrocyte differentiation. The authors used this biomechanical and biochemical combined strategy in an attempt to construct a physiological anisotropic meniscus. Their results showed that the cells expressed zonal-specific differences in the expression of the COL I and COL II, and also presented with a long-term chondroprotection of the knee joint when implanted into a rabbit model.

In short, relevant studies have brought hope on zonal meniscal reconstruction which indicate that more attempts should paid on biomaterials innovations, manufacturing technologies, biomechanical stimulation, and other novel techniques.

### Immune Compatibility and Tunability Studies

Polymeric materials science of the past focused on biocompatibility, including foreign body reactions (FBRs), degradability, and toxicity. However, current studies are aimed at solving one hurdle in terms of tissue engineering, that is, immune compatibility. In general, more advanced polymeric scaffolds are needed not only to fulfill the criteria of the present studies but also to regulate both the innate and adaptive immune responses.

Synthetic polymers, such as polyesters, are frequently used as cellular frameworks to support neotissue formation (Lee et al., 2019; Patel et al., 2019). For a long time, the immune compatibility of synthetic polymers focused on reducing the unwanted effects of FBR, fibrotic encapsulation, and toxicity (Chung et al., 2017). Numerous studies have confirmed that FBR may be a consistent process involved in neutrophil infiltration, monocyte/macrophage influx, fibroblast-mediated collagen and capillary bed formation, and the systemic response may also contribute to this process (Chung et al., 2017). On the other hand, natural polymers are manipulated not to induce an FBR response but to be a primary promoter for the regenerative process. For example, Ji’s and Lei’s groups found that glycidyl methacrylate-modified thermosensitive hydroxypropyl chitin hydrogels polarize macrophages both *in vitro* and *in vivo* at the cartilage defect site to accelerate a pro-regenerative process (Ji et al., 2020). It is believed that the bioactive motifs within ECM or ECM-like biological polymers can directly modulate the immune response (Rowley et al., 2019). For instance, the fibronectin sites FNI_1__0__–__1__2_ and FNII_1__–__5_ have been demonstrated to be able to modulate cellular responses in the regenerative cascade (Sawicka et al., 2015).

Apart from the composition of polymers, the factors involved in the immune response to biomaterials require further investigation. Interestingly, Veiseh et al. (2015) proved that spherical polymers (≥1.5 mm) in diameter triggered more severe fibrotic reactions than smaller particles or other shaped constructs, which indicated that the geometry and dimension of biomaterials are also vital impact factors of the FBR response. Currently, polymeric scaffolds are emerging as orchestrators to modulate the immune response that can influence tissue regeneration and repair processes (Chung et al., 2017). Recent research on biomaterial-mediated immunomodulation has two main directions. First, we focused on the modification of engineering scaffolds. A variety of surface chemical features have been studied, including the functional groups, surface charge, hydrophilicity, and molecular weight of the compound (Lee et al., 2019). Delivering anti-inflammatory drugs or cytokines is a second direction for engineered scaffolds to stimulate immunomodulation responses. For example, decellularized cartilage extracellular matrix has been decorated with the anti-inflammatory cytokine IL-4, and this modified scaffold promoted M2 macrophage polarization and osteochondral repair in a rat model (Tian et al., 2021). In addition, acellular cartilage matrix scaffolds functionalized with Wharton’s jelly mesenchymal stem cell-derived exosomes successfully reduced the inflammatory response in the joint cavity of a rabbit osteochondral model and improved cell proliferation, migration and polarization *in vitro*.

In summary, in recent decades, various biomaterial-mediated meniscal tissue engineering strategies have mainly focused on modulating the activity of different cells involved in meniscal regeneration to induce meniscal tissue formation. However, more attention should be given to the topic of biomaterial design to modulate the immune response and avoid undesirable inflammatory responses (Lee et al., 2019). To date, there are no systematic studies on the impact of immunomodulatory biomaterials on meniscal regeneration. We believe that this kind of engineered scaffold could have a profound impact on meniscal regeneration and associated patient recovery.

## Challenges and Future Prospects

Our increasing understanding of the crucial functions of the meniscus in knee homeostasis propels clinicians and scientists to treat meniscal tears with more promising strategies. A successful regenerative strategy for meniscal tissue engineering requires a profound understanding of the structure and composition of native menisci. First, the investigation and reconstruction of the structure-composition-function relationship remains a primary challenge of meniscal regeneration. The capability of the meniscus to withstand mechanical functions in terms of resistance to tension, compression and shear stress is mostly attributed to the collagen-proteoglycan meniscal matrix; however, complete reconstruction of this structure has not yet been attained. With decades of development of biomaterial science, a range of polymers spanning from natural to synthetic have been optimized in the search for meniscal scaffolds with favorable indications in animal studies. In this context, recent efforts have focused on meniscal tissue engineering, which relies on well-designed scaffolds for regeneration and replacement purposes. Engineered menisci with more elaborate design and better biomimicry have emerged along with a deepening understanding of the composition and architecture, cellular biology and biomechanics of the meniscus.

The optimal scaffold should be characterized by many biophysical and biochemical properties, as well as bioactivity, to guarantee an ECM-like microenvironment for cell survival and differentiation and restoration of anatomical and mechanical properties of native meniscus. Generally, selecting polymers remains a knotty problem since natural polymers present better biocompatibility and intrinsic bioactivity, whereas synthetic polymers have superior mechanical properties. However, single-polymer scaffolds are insufficient to mimic the meniscal composition and structure. To address this problem, in preliminary studies, natural and synthetic polymer combinations have been developed and have shown promising therapeutic results. More importantly, meniscal tissue is a connective tissue with zonal variations in mechanical properties, cellular composition and vascularization. Thus, zonal meniscal regeneration represents another major challenge since individual size and shape variations and stable implant fixation also present difficulties to be overcome. For this purpose, improvements in manufacturing technologies, especially bioreactors and 3D bioprinting, are set to overcome the challenges mentioned above. Bioreactors used in tissue engineering have been proved to provide efficient biomechanical stimulus and thus accelerate region-specific differentiation, demonstrated to be a beneficial way in zonal meniscal reconstruction. Alternatively, 3D bioprinting technologies can be used to manufacturing zonally variant meniscus. 3D bioprinting techniques can ideally help mimic the specific anatomical size and shape of native menisci, and the macro/microporous properties of these fiber-reinforced constructs are also beneficial for guiding ECM deposition and cell distribution. Indeed, advanced bioinks are capable of producing constructs with a composition resembling that of the native meniscus as closely as possible. Furthermore, 3D-printed scaffolds can be loaded with multiple cells and bioactive molecules within bioinks to further simulate dynamic regeneration *in vivo*. For all these reasons, 3D bioprinting represents one of the most promising strategies in meniscal tissue engineering, although the suboptimal ability of this approach to construct zone-specific scaffolds requires further improvement.

Second, considering the limited self-repair capacity of the meniscus, which is mainly due to a lack of blood vessels, nerves and lymphatic tissue, investigating the innate regeneration process of the meniscus is required (Li et al., 2020a). The top priority for meniscal tissue engineering is still the formation of neotissue at the site of injury, especially the avascular zone of the meniscus, which has no self-healing ability. In the natural healing process, once tissue is injured, endogenous stem cells respond to biochemical signals, migrate to damaged sites, differentiate into somatic cells and restore their morphology and function (Yang et al., 2020). Ideally, these reparative cells usually mobilize upon the provision of chemotactic gradients. However, a major concern for meniscal scaffolds is that almost all endogenous cells are hampered by the surrounding dense ECM, which hinders cell migration (Patel et al., 2019). One additional concern for meniscal scaffolds is limited infiltration into the center of the scaffold. Therefore, polymeric materials that are able to induce cell migration and provide a suitable microenvironment for cell adhesion and proliferation have been shown to be promising candidates for use in meniscal regeneration. For example, Zhang et al. (2016) demonstrated that a 3D-printed PCL scaffold with a mean pore size of 215 μm significantly enhanced seeded cell colonization and further improved meniscal regeneration. Aside from changing the scaffold porosity, the incorporation of chemotactic chemokines could be a promising avenue (Tarafder et al., 2019; Baek et al., 2020; Zhu et al., 2020).

**FIGURE 6:**
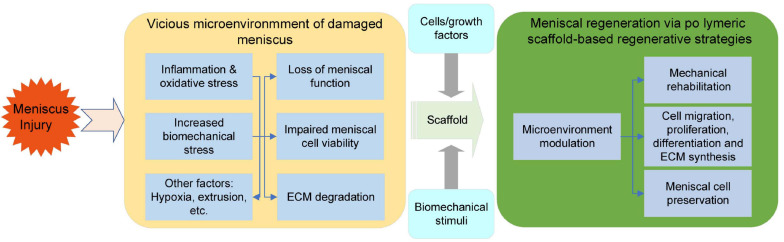
Hostile microenvironment of damaged meniscal tissue and the regenerative process initiated via polymeric scaffolds.

Apart from an amendable interfacial design and proper recruitment signals, relevant stimuli for regulating chondrogenesis are also needed. The discovery and exploration of types of biophysical and biochemical stimuli recently demonstrated promising contributions to meniscal scaffolds for meniscal repair. However, the meniscus exhibits spatial variations in cell phenotypes and components. Therefore, biofactor-functionalized scaffolds capable of spatiotemporal release hold great promise. A series of studies demonstrated that CTGF and TGF-β3 can induce zone-specific matrix formation *in vitro* and *in vivo* and may be a suitable combination for meniscal regeneration (Lee et al., 2014). Despite the type of material, biophysical properties, such as mechanical strength and matrix stiffness, can also influence cell fate (Wei et al., 2020). To mimic the development of neomeniscal tissue, the physical stimuli attributed to meniscal regeneration were proven by Zhang et al. (2019). In their study, a customized dynamic tension-compression loading system was developed to accelerate meniscal tissue formation *in vitro* and achieved anisotropic reconstruction of the meniscus *in vivo* (Zhang et al., 2019; Figure 5). This technique may inspire the future development and application of polymeric materials. Based on these findings, we believe that the optimization of materials, the selection of bioactive molecules and stimuli and the synergistic effect of their combinations as a bridge over the vicious cycle of chronic meniscal damage and support meniscal regeneration will most likely be future directions (Figure 6).

Finally, the success of polymeric biomaterials for meniscal regeneration depends on how well scaffolds interact with the *in vivo* local meniscal microenvironment and modulate the healing process. The immune response to biomaterials is another major barrier between successful application and scaffold implantation. Meniscal injury is characterized by the activation of inflammation and catabolism. For instance, synovitis after a meniscal tear often leads to mild to moderate inflammation within the joint and acts as a predictor of postmeniscectomy joint dysfunction (Riera et al., 2011; Scanzello et al., 2013). In recent years, we have gained a better understanding of the immunomodulatory effects of biomaterials in tissue regeneration and have paid more attention to designing and manufacturing “immune reprogramming” biomaterials rather than simply “immune friendly” biomaterials. Although previous studies have not yet extensively analyzed or experimented with immunomodulatory biomaterials in the meniscus, we believe that the current biomaterial-mediated immunomodulatory approaches that realize surface modification with bioactive factors will inevitably be beneficial for meniscal tissue engineering in the future.

Currently, we cannot obtain a tissue-engineered meniscus that meets all requirements and is functionalized as it is in its native state. However, a more profound understanding and the application of supportive polymeric materials in combination with other approaches to initiate endogenous meniscal regeneration would provide precious advances for meniscal regeneration (Figure 6).

## Conclusion

Treatment of meniscal injuries currently remains a challenge, which calls for an advanced treatment strategy based on an understanding of the pathophysiology of damaged tissue. There is an urgent clinical need for more applicable therapeutics that are able to recapitulate not only the native microarchitecture and components but also the spatiotemporal signaling cascades in the healing process of meniscal tissue. In this progress report, we highlight recent developments in the field of polymeric biomaterials science and associated manufacturing technology for the repair and regeneration of meniscal tissue. Further investigation is required to produce, select, and combine ideal polymeric biomaterials that are repeatable, non-toxic, non-immunogenic, biodegradable, and have the ability to provide biophysical and/or biochemical cues for the regeneration of meniscal tissue. The formulation of a hybrid polymeric scaffold aimed at providing an optimal microenvironment, mechanical support, and delivery bioactivities proved to be a synergistically advanced approach. In addition, current scaffold manufacturing technologies based on 3D printing are proving promising. At the same time, we envision that the zonal meniscal reconstruction and immune compatibility of the scaffold are also vital factor that should be investigated in meniscal tissue engineering. In short, future exploration should be focused on the development of optimum biomaterials and advanced scaffold manufacturing technologies as well as the implications of biomaterial-directed immunomodulation that orchestrates the pro-regenerative process and finally promotes the anisotropic reconstruction of the native, predamaged meniscus.

## Author Contributions

HL and PL designed the manuscript and wrote part of it. ZYa wrote the part of the manuscript with insights from all other authors. LF and CG conceptualized and generated Tables 1–4. TZ, WC, ZL, YP, and FC conceptualized and generated Figures 1, 2, 4, 6. XS, ZYu, SL, and QG revised and finalized the manuscript. All authors read and approved the final manuscript, contributed to manuscript revision, and read and approved the submitted version.

## Conflict of Interest

The authors declare that the research was conducted in the absence of any commercial or financial relationships that could be construed as a potential conflict of interest.
